# Microvascular lung vessels obstructive thromboinflammatory syndrome in patients with COVID-19: Insights from lung intravascular optical coherence tomography

**DOI:** 10.3389/fmed.2023.1050531

**Published:** 2023-02-16

**Authors:** Ludhmila Abrahão Hajjar, Marco B. Ancona, Roberto Kalil Filho, Moreno Tresoldi, José Guilherme Caldas, Giacomo Monti, Francisco Cesar Carnevale, Francesco De Cobelli, André Moreira de Assis, Fabio Ciceri, Giovanni Landoni, Jouke Dijkstra, Francesco Moroni, Alexandre Antônio Cunha Abizaid, Fernanda Willemann Ungaretti, Maria José Carvalho Carmona, Daniel De Backer, Carlos Eduardo Pompilio, Fábio S. de Britto, Carlos M. Campos, Alberto Zangrillo, Matteo Montorfano

**Affiliations:** ^1^Heart Institute Faculdade de Medicina da Universidade de São Paulo, São Paulo, Brazil; ^2^Interventional Cardiology Unit, IRCCS San Raffaele Scientific Institute, Milan, Italy; ^3^Unit of General Medicine and Advanced Care, IRCCS San Raffaele Scientific Institute, Milan, Italy; ^4^Department of Interventional Neuroradiology, Faculdade de Medicina da Universidade de São Paulo, São Paulo, Brazil; ^5^Anesthesia and Intensive Care Department, IRCCS San Raffaele Scientific Institute, Milan, Italy; ^6^Interventional Radiology Department, Radiology Institute, Faculdade de Medicina da Universidade de São Paulo, São Paulo, Brazil; ^7^Radiology Department, IRCCS San Raffaele Scientific Institute, Milan, Italy; ^8^School of Medicine, Vita-Salute San Raffaele University, Milan, Italy; ^9^Department of Hematology and Bone Marrow Transplantation, IRCCS San Raffaele Scientific Institute, Milan, Italy; ^10^Division of Image Processing, Department of Radiology, Leiden University Medical Center, Leiden, Netherlands; ^11^Discipline of Anesthesiology, Hospital das Clínicas da Faculdade de Medicina da Universidade de São Paulo, São Paulo, Brazil; ^12^Department of Intensive Care, CHIREC Hospitals, Université Libre de Bruxelles, Brussels, Belgium; ^13^Department of Intensive Care, Hospital das Clínicas da Faculdade de Medicina da Universidade de São Paulo, São Paulo, Brazil

**Keywords:** COVID-19, MicroClots, OCT, thrombo-inflammatory syndrome, D-dimer

## Abstract

**Background:**

Microvascular lung vessels obstructive thromboinflammatory syndrome has been proposed as a possible mechanism of respiratory failure in COVID-19 patients. However, it has only been observed in post-mortem studies and has never been documented *in vivo*, probably because of a lack of CT scan sensitivity in small pulmonary arteries. The aim of the present study was to assess the safety, tolerability, and diagnostic value of optical coherence tomography (OCT) for the assessment of patients with COVID-19 pneumonia for pulmonary microvascular thromboinflammatory syndrome.

**Methods:**

The COVID-OCT trial was a multicenter, open-label, prospective, interventional clinical study. Two cohorts of patients were included in the study and underwent pulmonary OCT evaluation. Cohort A consisted of patients with COVID-19 with a negative CT scan for pulmonary thrombosis and elevated thromboinflammatory markers (D-dimer > 10,000 ng/mL or 5,000 < D-dimer < 10,000 ng/mL and one of: C-reactive Protein > 100 mg/dL, IL-6 > 6 pg/mL, or ferritin > 900 ng/L). Cohort B consisted of patients with COVID-19 and a CT scan positive for pulmonary thrombosis. The primary endpoints of the study were: (i) to evaluate the overall safety of OCT investigation in patients with COVID-19 pneumonia, and (ii) to report on the potential value of OCT as a novel diagnostic tool for the diagnosis of microvascular pulmonary thrombosis in COVID-19 patients.

**Results:**

A total of 13 patients were enrolled. The mean number of OCT runs performed in each patient was 6.1 ± 2.0, both in ground glass and healthy lung areas, achieving a good evaluation of the distal pulmonary arteries. Overall, OCT runs identified microvascular thrombosis in 8 patients (61.5%): 5 cases of red thrombus, 1 case of white thrombus, and 2 cases of mixed thrombus. In Cohort A, the minimal lumen area was 3.5 ± 4.6 mm^2^, with stenosis of 60.9 ± 35.9% of the area, and the mean length of thrombus-containing lesions was 5.4 ± 3.0 mm. In Cohort B, the percentage area obstruction was 92.6 ± 2.6, and the mean thrombus-containing lesion length was 14.1 ± 13.9 mm. No peri-procedural complications occurred in any of the 13 patients.

**Conclusion:**

OCT appears to be a safe and accurate method of evaluating the distal pulmonary arteries in hospitalized COVID-19 patients. Here, it enabled the first *in vivo* documentation of distal pulmonary arterial thrombosis in patients with elevated thromboinflammatory markers, even when their CT angiogram was negative for pulmonary thrombosis.

**Clinical trial registration:**

ClinicalTrial.gov, identifier NCT04410549.

## Introduction

Severe acute respiratory syndrome coronavirus 2 (SARS-CoV-2) infection represents a pandemic emergency. The clinical course of SARS-CoV-2 infection often meets the criteria for acute respiratory distress syndrome (ARDS), with a high mortality rate.

The worsening of lung function in patients infected with SARS-CoV-2 could be driven by a host immune response. SARS-CoV-2 replication in lung epithelial cells causes cellular damage with release of pro-inflammatory alarmins. The subsequent complement system activation causes local release of pro-inflammatory cytokines, with severe collateral tissue injury; alveolar epithelial and vascular endothelial cell damage, together with microvascular thrombosis, can thus occur. This particular form of ARDS can lead to a progressive worsening of ventilation/perfusion imbalances and a loss of hypoxic vasoconstriction reflexes, with a marked component of microvascular pulmonary thrombosis, as suggested by elevated levels of lactate dehydrogenase and D-dimer. In the late stages of the disease, the endothelial damage progression with microvascular thrombosis can spread locally in the lung, and the systemic inflammatory reaction is extended to involve the microvascular bed of the kidneys, brain, and other vital organs (1). MicroCLOTS (microvascular COVID-19 lung vessels obstructive thromboinflammatory syndrome) has been proposed as a working hypothesis on the nature of this atypical ARDS (1), involving a new mechanism of lung damage: alveolar endothelial damage leading to progressive endothelial pulmonary syndrome and microvascular thrombosis. In fact, the rate of thromboembolic events in COVID-19 patients appears to be non-negligible (2), and prophylactic utilization of low-molecular-weight heparin (LMWH) is indicated in hospitalized patients (3).

In an effort to stratify COVID-19 patients, several biomarkers are under investigation for their potential in better determining the risk of thromboembolic events and in identifying those patients who could benefit from prophylactic therapy with LMWH. Among other possible biomarkers, D-dimer is often elevated in COVID-19 patients (4, 5) and should be regarded as the most important parameter for stratification in terms of thromboembolic risk; it should be used together with other inflammatory markers, such as C-reactive protein, interleukin 6 (IL-6), and ferritin. Nevertheless, a non-negligible proportion of patients with COVID-19 pneumonia present with a high D-dimer level along with negative computed tomography (CT) angiography for pulmonary embolism. Although these patients could be experiencing pulmonary microvascular thrombosis (MicroCLOTS) (1), this hypothesis has never been proven, and a more potent anticoagulation regimen is currently not being routinely utilized. A more sensitive diagnostic technique than the CT scan for the evaluation of small pulmonary arteries could theoretically allow the detection of MicroCLOTS, thus justifying a more potent anticoagulant regimen.

Optical coherence tomography (OCT) is an invasive imaging technique based on the use of a near-infrared light source, with a resolution of 10–20 μm (6). OCT has shown good correlation with histology in the evaluation of pulmonary artery morphology (7), in the characterization of distal-type chronic thromboembolic pulmonary hypertension (8), and in providing guidance for treatment of the latter via percutaneous transluminal pulmonary angioplasty (9). However, there are no data on OCT detection of MicroCLOTS in COVID-19 patients.

The aim of the present study was to evaluate the presence of microvascular pulmonary thrombosis via intravascular OCT in patients with COVID-19 and high D-dimer levels, and to compare this method with CT angiography for pulmonary thrombosis.

## Materials and methods

The COVID-OCT trial (NCT04410549) was an open-label, multicenter, prospective, interventional clinical study of the safety, tolerability, and potential diagnostic value of OCT for assessment of pulmonary microvascular obstructive thromboinflammatory syndrome in patients with COVID-19 pneumonia.

A complete list of the inclusion and exclusion criteria for this study is provided in the Supplementary material. Two convenience sample cohorts of patients were prospectively recruited. Cohort A consisted of patients with severe COVID-19 and suspected MicroCLOTS, and with a negative CT scan for pulmonary thrombosis. Cohort B consisted of patients with COVID-19 and Suspected MicroCLOTS was established via the presence of increased thromboinflammatory markers (D-dimer > 10,000 ng/mL, or 5,000 < D-dimer < 10,000 ng/mL and one of: C-reactive protein (CRP) > 100 mg/dL, IL-6 > 6 pg/mL, or ferritin > 900 ng/L).

### OCT acquisition and analysis

Following 6 French femoral vein echo-guided puncture, selective pulmonary artery cannulation and angiography was performed. The choice of pulmonary arteries to be cannulated was driven by the “ground glass” area on the patient’s CT scan. A 0.014” floppy wire was advanced distally in the pulmonary artery and the OCT catheter (Dragonfly OPTIS Imaging Catheter, Abbott Vascular, Santa Clara, CA) was introduced. In order to remove all the blood and to obtain clear images, iodinated contrast media was infused at a flow rate of 5 mL/s over 4 s at a pressure of 400 psi using an automated injector (Acist, Eden Prairie, Minnesota). OCT acquisition was performed using the OPTIS™ imaging system (Abbott Vascular, Redwood, United States). OCT images were acquired at a rate of 20 frames/s with a pullback speed of 20 mm/s. All recordings were performed according to the recommended procedure for each OCT system. The pulmonary artery analysis was performed at 1-mm intervals. Light intensity analysis was performed using dedicated software (QCU-CMS v4.69 research version, Leiden, Netherlands). Raw images in original polar format were used to ensure that interpolation, dynamic range compression, or other facets of image processing would not alter the signal and bias the analysis. The lumen contour and thrombus contours were delineated automatically, with manual corrections where appropriate. Based on the original image, dedicated software was used to carry out fully automatic computation of three components of light property analysis: (a) light intensity, (b) light attenuation, and (c) backscatter.

The study was conducted in compliance with the Declaration of Helsinki and the Good Medical Practice standards. All patients provided written informed consent to take part in the study. The study was reviewed and approved by the local ethics committees (registration number 93/INT/2020) and did not receive any financial support.

### Data analysis

Continuous variables are expressed as means (standard deviation) or medians [interquartile range (IQR)], while categorical variables are reported in the form of counts and percentages. The normality assumption was verified using the Shapiro–Wilk test. The small sample did not allow for further statistical analyses. Statistical analyses were carried out using IBM SPSS Statistics 24 (SPSS, Chicago, Illinois).

## Results

**FIGURE 1:**
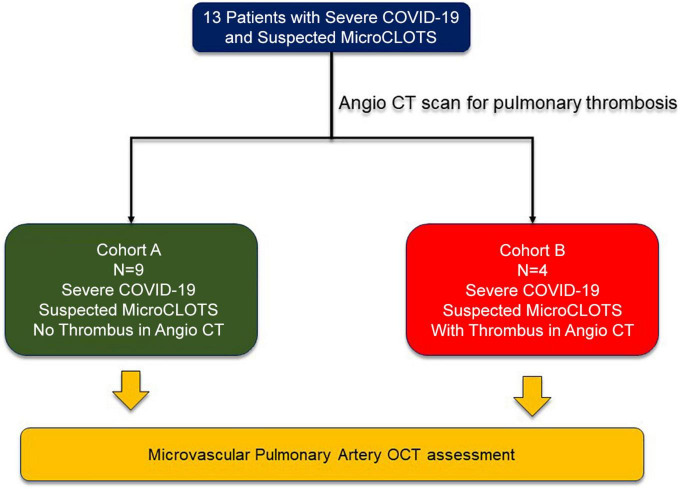
Flowchart of patients included in the present study.

A total of 13 patients across two centers (San Raffaele Scientific Institute, Milan, Italy and Incor Heart Institute, University of São Paulo, Brazil) were enrolled in the study between June 2020 and March 2021 (Figure 1). Nine patients had a negative CT scan for thrombosis (Cohort A) and 4 patients had a positive CT scan for pulmonary thrombosis (Cohort B). Detailed information on the clinical characteristics of the study population is provided in the Supplementary Tables 1, 2.

### Procedural safety

No peri-procedural complications occurred in any of the 13 patients. According to BARC definitions (10), there were no major (BARC Type 3) or life-threatening (BARC Type 5) incidents of bleeding related to the procedure. One patient experienced acute kidney injury several days after the procedure, most likely related to septic shock. The primary endpoint of procedural safety and accuracy was thus achieved in all patients. The small sample size of this pilot study did not allow for analysis of the correlation between OCT findings and pulmonary hypertension, right ventricular dysfunction, or standard inflammatory, coagulation, and tissue damage markers.

The mean age among patients included in Cohort A was 61.1 ± 13.8 years; seven of the nine patients (77.7%) were male. The mean time between onset of symptoms and admission was 8.5 ± 4.4 days. Six patients (66.6%) were on invasive mechanical ventilation and one patient was on non-invasive mechanical ventilation, while one patient was receiving only oxygen. The mean PaO2/FiO2 was 184 ± 59 mmHg. According to the inclusion criteria, D-dimer values were elevated in the study population (median: 7070 ng/ml; interquartile range: 4,017 – 24,001 ng/ml), as were C-reactive protein levels. Myocardial damage was not significant, with only a mild increase in troponin T occurring in most cases and all patients having normal left and right ventricular systolic function; only one patient presented with pulmonary hypertension. Ground-glass opacities were observed at CT scan in all patients.

In Cohort B, the mean age was 64.5 ± 8.1; the mean time between onset of symptoms and hospital admission was 9.5 ± 3.5 days. Three patients (75%) were on invasive mechanical ventilation, while one patient was on non-invasive mechanical ventilation. The mean PaO2/FiO2 was 134 ± 39 mmHg. All patients included in Cohort B had a positive CT scan for pulmonary embolism together with ground glass opacities and consolidation areas. Interestingly, none of the patients enrolled in Cohort B had signs of deep vein thrombosis.

### OCT findings

**FIGURE 2:**
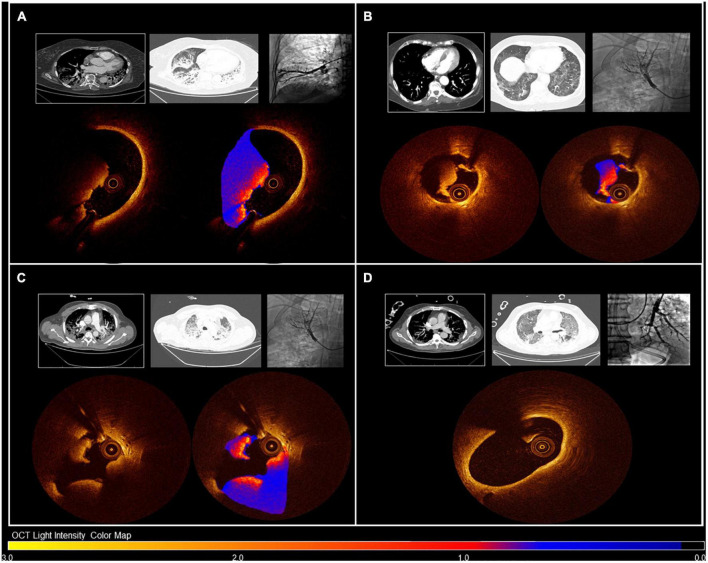
OCT images of patients with distinct pulmonary microthrombosis presentation. Images are color-coded for OCT assessment of light intensity: darker colors indicate light attenuation, and lighter colors indicate areas of high light-intensity material. Panel **(A)**: Patient 4, Cohort B. CT scan was positive for pulmonary embolism. OCT confirmed distal pulmonary thrombosis with red thrombus. Panel **(B)**: Patient 3, Cohort A. CT scan was negative for pulmonary embolism. OCT showed distal pulmonary thrombosis with white thrombus. Panel **(C)**: Patient 2, Cohort B. CT scan was positive for pulmonary embolism. OCT confirmed distal pulmonary thrombosis with mixed thrombus. Panel **(D)**: Patient 1, Cohort A. CT scan was negative for pulmonary embolism. OCT did not show any distal pulmonary thrombosis.

The mean number of OCT runs performed in each patient was 6.1 ± 2.0, achieving good evaluation of the distal pulmonary arteries. Figure 2 shows examples of pulmonary OCT findings. Pulmonary angiography with OCT was successfully performed in all patients. Overall, OCT runs identified microvascular thrombosis in 8 patients (61.5%): 5 cases of red thrombus, 1 case of white thrombus, and 2 cases of mixed thrombus.

Among the nine patients without thrombus according to CT angiogram (Cohort A), distal pulmonary thrombosis was documented via OCT in four patients (44.4%). A detailed list of OCT findings in the four patients with positive OCT is provided in Table 1. The mean reference segment diameter and area were 2.9 ± 0.6 mm and 6.8 ± 2.9mm^2^, respectively. At thrombus regions, mean vessel diameter was 1.8 ± 1.2 mm. The minimal lumen area was 3.5 ± 4.6mm^2^, with stenosis of 60.9 ± 35.9% of the area, and the mean length of thrombus-containing lesions was 5.4 ± 3.0 mm. In two patients, the documented thrombus was red; in one patient, it was white; and in one patient, mixed white and red thrombus was observed. Interestingly, deep vein thrombosis was documented in only one of the nine patients enrolled in Cohort A (11.1%).

**TABLE 1:** OCT findings for arteries with thrombus-containing lesions in patients with negative CT scan for pulmonary thrombosis: Cohort A.

Variable	Patient 3A	Patient 4A	Patient 6A	Patient 7A	Mean ± SD
**Reference segment**					
Reference area (mm^2^)	5.2	11.3	5.7	5.2	6.8 ± 2.9
Mean reference diameter (mm)	2.6	3.8	2.7	2.6	2.9 ± 0.6
**Region of Interest**					
Thrombus type	White	Mixed	Red	Red	
Thrombus-containing lesion length (mm)	5.2	2.0	9.4	5.2	5.4 ± 3.0
MLA (mm^2^)	1.5	10.4	1.3	0.7	3.5 ± 4.6
Area obstructed (%)	71.3	7.9	77.5	87.1	60.9 ± 35.9
Minimal diameter (mm)	1.0	2.7	0.9	0.8	1.3 ± 0.9
Maximal diameter (mm)	1.8	4.0	2.0	1.2	2.2 ± 1.2
Mean diameter (mm)	1.4	3.6	1.3	0.9	1.8 ± 1.2
Mean circumferential thrombus angle (degrees)	165	36	160	138	124.7 ± 60.3
Max circumferential thrombus angle (degrees)	145	17	166	178	126.5 ± 74.3

SD: standard deviation.

In patients with documented pulmonary embolism according to CT angiogram (Cohort B), distal pulmonary thrombosis was documented via OCT in all patients (3 cases of red thrombus and 1 case of mixed thrombus). The minimal lumen area was 1.6 ± 0.6mm^2^, with a mean percentage area obstruction of 92.6 ± 2.6, and the mean thrombus-containing lesion length was 14.1 ± 13.9 mm (Table 2).

**TABLE 2:** OCT findings for arteries with thrombus-containing lesions in patients with positive CT scan for pulmonary thrombosis: Cohort B.

Variable	Patient 1B	Patient 2B	Patient 3B	Patient 4B	Mean ± SD
**Reference segment**					
Reference area (mm^2^)	15.3	NA	24.5	15.5	18.4 ± 5.3
Mean reference diameter (mm)	4.4	NA	5.6	4.4	4.8 ± 0.69
**Region of Interest**					
Thrombus type	Red	Mixed	Red	Red	
Thrombus-containing lesion length (mm)	35	8.4	5.8	7.3	14.1 ± 13.9
MLA (mm^2^)	0.9	2.3	1.4	1.6	1.6 ± 0.6
Area obstructed (%)	94.1	NA	94.1	89.6	92.6 ± 2.6
Minimal diameter (mm)	1.2	0.5	1.1	0.6	0.9 ± 0.4
Maximal diameter (mm)	2.3	2.5	1.7	2.8	2.3 ± 0.5
Mean diameter (mm)	1.6	2.2	1.3	1.4	1.6 ± 0.4
Mean circumferential thrombus angle (degrees)	NA	245.3	319.6	121.9	228.9 ± 99.9
Max circumferential thrombus angle (degrees)	NA	272.9	334.8	137.3	248.3 ± 101.0

NA, not available; SD, standard deviation.

### Performance of CT scan for MicroCLOTS in COVID-19 patients

CT scan had a sensitivity of 50% and specificity of 100%, for a positive predictive value of 100% and negative predictive value of 55.56% in detecting MicroCLOTS in COVID-19 patients.

## Discussion

The main findings of the present study can be summarized as follows: (1) microvascular thrombosis was frequently found in COVID-19 patients, even when angiotomography was negative for it; (2) the tolerability and diagnostic value of OCT for the assessment of pulmonary microvascular obstructive thromboinflammatory syndrome in patients with COVID-19 pneumonia were confirmed.

The primary endpoint of the present study was procedural safety, tolerability, and accuracy; this was achieved in all patients. Among the nine patients with a CT scan negative for pulmonary embolism, OCT detected distal pulmonary thrombus in four patients (44.4%). Furthermore, OCT was able to detect distal pulmonary thrombus in all patients who already had a positive CT scan for pulmonary embolism, even in areas of the lungs where the CT scan had not detected any thrombus.

Hypercoagulability (11) appears as a common clinical manifestation of COVID-19: D-dimer is frequently elevated (4) and thromboembolic events appear to be frequent. Among hospitalized COVID-19 patients, pooled incidence rates of 21, 15, and 27% have been reported for venous thromboembolic events (VTEs), pulmonary embolism (PE), and deep vein thrombosis (DVT), respectively (12). Furthermore, a fourfold increase has been reported in VTE rate among patients being cared for in intensive care units compared with those in non-intensive care unit settings (28% vs. 7%) (13). It has therefore been hypothesized that inflammation associated with SARS-CoV-2 infection leads to “Covid-19–related coagulopathy”, which is responsible for the observed increase in thrombosis (14).

Pulmonary microvascular thrombosis has been proposed as a possible mechanism of respiratory failure in COVID-19 patients. Given the rate of suspected microvascular thrombosis and the increased rate of thrombotic complication in hospitalized patients with Covid-19, several observational studies have investigated the use of anticoagulant therapy; these have shown increased survival in hospitalized patients treated with this therapy (15). Several randomized controlled trials are currently in progress to evaluate the effectiveness of anticoagulant therapy in patients with COVID-19 disease (16, 17). However, pulmonary microvascular thrombosis has only been demonstrated in post-mortem studies (18, 19) and has never been documented *in vivo*, probably because of the lack of sensitivity of CT scans in small pulmonary arteries.

This is the first *in vivo* documentation of distal pulmonary artery thrombosis in COVID-19 patients. OCT was able to detect small distal pulmonary artery thrombosis in patients with a negative CT scan for pulmonary embolism and elevated thromboinflammatory markers (i.e., high D-dimer, CRP, IL-6, or ferritin). The findings of the present study raise the possibility of a link between CT glassy infiltrates and microvascular pulmonary thrombosis in this scenario.

OCT acquires longitudinal sequences of cross-sectional images (at a rate of up to 100 frames/s) in a blood-free environment, resulting in sharp definition of the border between lumen and vessel wall. It is routinely used in percutaneous coronary intervention (PCI) to better characterize vessel anatomy, as well as for ascertainment of full stent deployment and expansion (20). Before the COVID-19 pandemic, OCT had already been used to confirm pulmonary artery thrombosis (21–24) used OCT to evaluate three patients who were strongly suspected for peripheral pulmonary artery thrombosis but had negative CT scans for pulmonary embolism. As in our study, thrombi were observed in most of the vessels imaged in these patients. Red and white thrombi could be differentiated according to features of the thrombus in OCT images. Following anticoagulation treatment, the patients’ symptoms and hypoxemia improved. Repeated OCT imaging showed that most thrombi had disappeared or become smaller.

These findings confirm the working hypothesis of MicroCLOTS and reinforce the need for randomized trials evaluating anticoagulant therapy in COVID-19 patients, especially if an increase in thromboinflammatory markers is present.

The study has a number of limitations. First, only a small number of participants were enrolled. Second, the invasiveness of the procedure limits the applicability of pulmonary OCT evaluation in clinical settings. Finally, OCT sensitivity in the detection of distal pulmonary artery thrombosis could have been limited by the OCT catheter itself: in fact, the OCT evaluation starts 3 cm proximal to the tip of the catheter, thus limiting evaluation of the more distal and smaller pulmonary arteries.

Future studies should validate this technique in a larger population in order to confirm the MicroCLOTS hypothesis and provide further evidence supporting the use of anticoagulant therapy in hospitalized COVID-19 patients.

## Conclusion

OCT appears to be an accurate method of evaluating the distal pulmonary arteries in hospitalized COVID-19 patients, allowing the first *in vivo* documentation of distal pulmonary arterial thrombosis in patients with a negative CT scan for pulmonary embolism and elevated thromboinflammatory markers. Further studies are needed to assess the efficacy of antithrombotic therapy in hospitalized patients with COVID-19.

## Data availability statement

The raw data supporting the conclusions of this article will be made available by the authors, without undue reservation.

## Ethics statement

The studies involving human participants were reviewed and approved by the Ethical Committee of Heart Institute Faculdade de Medicina da Universidade de São Paulo, São Paulo, Brazil and of San Raffaele Scientific Institute, Milan, Italy.

## Author contributions

MA, GL, MM, LH, MT, FCi, and AZ: study concept and design. RF, JC, GM, FCa, FD, AM, CC, JD, AA, FW, MC, FB, and MT: data acquisition. LH, MA, GL, CC, and FM: manuscript drafting. MM, GL, AA, and AZ: final approval of the manuscript. All authors contributed to the article and approved the submitted version.
