# Supplementary material for: Microvascular lung vessels obstructive thromboinflammatory syndrome in patients with COVID-19: Insights from lung intravascular optical coherence tomography
Source: Front Med (Lausanne). 2023 Feb 16;10:1050531. doi: 10.3389/fmed.2023.1050531 (PMC9978141; doi:10.3389/fmed.2023.1050531)
Supplement: Supplementary file 1 [file Data_Sheet_1.docx]

**Microvascular lung vessels obstructive thromboinflammatory syndrome in patients with COVID-19: insights from angiotomography and optical coherence tomography**

Ludhmila Abrahão Hajjar MD^1^, Marco B Ancona MD^2^, Roberto Kalil Filho MD^1^, Moreno Tresoldi MD^3^, José Guilherme Caldas MD^4^, Giacomo Monti MD^5^, Francisco Cesar Carnevale MD^6^, Francesco De Cobelli MD^7,8^, André Moreira de Assis MD^6^, Fabio Ciceri MD^8,9^, Giovanni Landoni MD^5,8^, Jouke Dijkstra PhD^10^, Francesco Moroni, MD^2^, Alexandre Antônio Cunha Abizaid MD, PhD^1^, Fernanda Willemann Ungaretti MD^1^, Maria José Carvalho Carmona MD^11^, Daniel De Backer, MD^12^; Carlos Eduardo Pompilio, MD, PhD^13^; Fábio S. de Britto Jr MD^1^,PhD; Carlos M. Campos MD, PhD^1^, Alberto Zangrillo MD^5,8^ and Matteo Montorfano MD^2^

1 Heart Institute Faculdade de Medicina da Universidade de São Paulo, São Paulo-SP, Brazil

2 Interventional Cardiology Unit, IRCCS San Raffaele Scientific Institute, Milan, Italy

3 Unit of General Medicine and Advanced Care, IRCCS San Raffaele Scientific Institute, Milan, Italy

4 Department of Interventional Neuroradiology, Faculdade de Medicina da Universidade de São Paulo, São Paulo SP, Brazil

5 Anesthesia and Intensive Care Department, IRCCS San Raffaele Scientific Institute, Milan, Italy

6 Interventional Radiology Department, Radiology Institute, Faculdade de Medicina da Universidade de São Paulo, São Paulo SP, Brazil

7 Radiology Department, IRCCS San Raffaele Scientific Institute, Milan, Italy

8 Vita-Salute San Raffaele University, Milan, Italy

9 Department of Hematology and Bone marrow Transplantation, IRCCS San Raffaele Scientific Institute, Milan, Italy

10 Division of Image Processing, Department of Radiology, Leiden University Medical Center, Leiden, The Netherlands

11 Discipline of Anesthesiology, Hospital das Clínicas da Faculdade de Medicina da Universidade de São Paulo, São Paulo SP, Brazil

12 Department of Intensive Care, CHIREC Hospitals, Université Libre de Bruxelles, Brussels, Belgium

13 Department of Intensive Care, Hospital das Clínicas da Faculdade de Medicina da Universidade de São Paulo, São Paulo SP, Brazil

**Inclusion Criteria (Cohort A)**

- Age > 18
- Severe pulmonary coronavirus disease 19 (COVID 19) with suspect for MicroCLOTS (microvascular COVID-19 lung vessels obstructive thromboinflammatory syndrome) **AND**
- Contrast CT scan negative for pulmonary thrombosis **AND**
- D-Dimer > 10 mcg/mL **OR**
- 5 < D-dimer < 10 mcg/mL and either C Reactive Protein (CRP) > 100 mg/dL or IL-6 > 6 pg/mL or ferritin > 900 ng/L

**Inclusion Criteria (Cohort B)**

- Age > 18
- Severe pulmonary coronavirus disease 19 (COVID 19) with suspect for MicroCLOTS (microvascular COVID-19 lung vessels obstructive thromboinflammatory syndrome) **AND**
- Contrast CT scan positive for pulmonary thrombosis

**Exclusion Criteria**

- Pregnancy or breastfeeding
- Known allergy to iodinated contrast dye
- Hemodynamic instability
- Glomerular Filtration rate < 30 ml/min
- Active bleeding or absolute contraindication to anticoagulant therapy

**Online Table 1. Baseline and Clinical Characteristics of Patients with CT scan negative for pulmonary embolism - Cohort A**

| Characteristics | Reference Value of Range | Patient 1 | Patient 2 | Patient 3 | Patient 4 | Patient 5 | Patient 6 | Patient 7 | Patient 8 | Patient 9 | Mean ± SD |
| --- | --- | --- | --- | --- | --- | --- | --- | --- | --- | --- | --- |
| Age -- yr |  | 60 | 38 | 69 | 76 | 45 | 49 | 74 | 67 | 72 | 64.5 ± 8.1 |
| Gender |  | male | male | female | male | male | female | male | male | male |  |
| Race |  | white | white | white | white | white | brown | brown | white | white |  |
| Medical history |  |  |  |  |  |  |  |  |  |  |  |
| Conditions |  | Former smoker | no | Former smoker | paroxysmal atrial fibrillation | no | obesity | coronary artery disease | coronary artery disease; hypertension | diabetes mellitus |  |
|  |  | coronary artery disease |  | hypertension |  |  | hypertension |  | rheumatoid arthritis |  |  |
|  |  |  |  | diabetes mellitus |  |  |  |  | prostate cancer |  |  |
| Time of symptoms onset at admission -- days |  | 10 | 10 | 16 | 10 | 7 | 10 | 15 | 3 | 2 | 9.5 ± 3.1 |
| Symptoms |  |  |  |  |  |  |  |  |  |  |  |
| Dyspnea / shortness of breath |  | yes | yes | yes | yes | yes | yes | yes | yes | yes |  |
| Dry cough |  | no | yes | yes | yes | yes | yes | no | yes | no |  |
| Fever |  | yes | no | no | no | no | yes | yes | no | no |  |
| Headache |  | yes | no | no | no | no | yes | no | no | no |  |
| Myalgia / arthralgia |  | yes | no | no | no | no | no | yes | no | yes |  |
| Fatigue |  | no | no | yes | yes | no | no | no | yes | yes |  |
| Type of Mechanical ventilation |  | Invasive | Invasive | Invasive | Invasive | Invasive | Invasive | Invasive | Non Invasive | No |  |
| Mechanical ventilation -- days |  | 10 | 4 | 6 | 2 | 3 | 3 | 3 | 3 | 0 | 3.8± 2.8 |
| PaO2/FiO2 ratio (mmHg) |  | 152 | 150 | 251 | 155 | 133 | 182 | 152 | 150 | 300 | 180.5 ± 56.6 |
| Anticoagulation |  | prophylatic | prophylatic | terapeuthic | prophylatic | prophylatic | terapeuthic | prophylatic | prophylatic | prophylatic |  |
| Laboratory values |  |  |  |  |  |  |  |  |  |  |  |
| D-dimer (ng/ml) § | < 500 | 5795 | 2239 | 41584 | 28349 | 19654 | 1660 | 8855 | 6970 | 7070 | 13575 ± 13596 |
| C-Reactive protein (mg/liter) | < 5 | 326 | 102 | 335 | 190 | 339 | 162.7 | 227.8 | 361.3 | 57.7 | 233.2 ± 112.7 |
| Ferritin (ng/ml) | 13 - 150 | 2731 | 2289 | 1684 | 2166 | NA | 583 | NA | 6302 | 373 | 2304 ± 1968 |
| Interleukin 6 (pg/ml) | ≤ 7 | NA | 4.1 | NA | NA | NA | 4.4 | NA | 54.5 | 54.8 | 29.4 ± 29.9 |
| Platelet count (10^3^/mm3) | 150 - 400 | 254 | 302 | 333 | 215 | 221 | 261 | 298 | 187 | 139 | 245.5 ± 61.5 |
| Prothrombin time (INR) | 0.95 - 1.2 | 1.03 | 1.01 | 0.91 | 0.95 | 0.93 | 1.07 | 1.27 | 1.02 | 1.00 | 1.0 ± 0.1 |
| Partial Thromboplastin time (sec) | 25.1 - 36.5 | 28.1 | 23.9 | 69.4 | 23 | 25 | 55.7 | 28.6 | 30.1 | 28.5 | 34.7 ± 16.3 |
| White-cell count (10^3^/mm3) | 4 - 11 | 6.7 | 8.9 | 15.4 | 10.9 | 5.6 | 3.9 | 7.3 | 6.3 | 4.2 | 7.7 ± 3.6 |
| Lymphocytes (10^3^/mm3) | 1.5-3.55 | 0.4 | 0.7 | 0.7 | 0.5 | 0.4 | 0.5 | 0.4 | 0.6 | 0.4 | 0.5 ± 0.1 |
| Hemoglobin (g/dl) | >13 | 10.3 | 11.5 | 11.6 | 12.7 | 10.1 | 9.5 | 11.9 | 11.9 | 13.8 | 11.4 ± 1.3 |
| Lactic dehydrogenase (U/liter) | 135 - 225 | 828 | 550 | 598 | 424 | 510 | 676 | 710 | 631 | 321 | 583 ± 153 |
| Protein Kinase (U/liter) | <190 | 332 | 605 | 869 | 384 | 2418 | 913 | 1657 | 54 | NA | 904 ± 781 |
| Troponin T (ng/ml) | ≤ 0.014 | 0.008 | 0.085 | 0.364 | 0.108 | 0.008 | 0.561 | 0.192 | NA | NA | 0.17 ± 0.17 |
| NT-proBNP (pg/ml) | <125 | 812 | 599 | 3137 | 649 | NA | 1397 | NA | NA | NA | 1318 ± 1064 |
| Creatinine (mg/dl) | 0.5 - 1.2 | 0.63 | 0.7 | 1.14 | 1.42 | 0.69 | 0.8 | 0.8 | 0.65 | 0.76 | 0.84 ± 0.26 |
| Total bilirrubin (mg/dl) | 0.2 - 1.0 | 1.06 | 1.12 | 0.74 | 1.37 | 0.44 | 0.63 | 0.53 | 0.88 | 0.27 | 0.78 ± 0.35 |
| Echocardiographic findings |  |  |  |  |  |  |  |  |  |  |  |
| LV ejection fraction (%) |  | 64 | 60 | 60 | 62 | 60 | 62 | 56 | NA | NA | 60.5 ± 2.5 |
| LV diastolic disfunction |  | impaired relaxation | no | no | no | no | no | no | NA | NA |  |
| RV Disfunction |  | no | no | no | no | no | no | no | NA | NA |  |
| Pulmonary hypertension |  | NA | NA | no | yes | no | no | no | NA | NA |  |
| CTA of chest findings |  |  |  |  |  |  |  |  |  |  |  |
| Pulmonary thromboembolism |  | no | no | no | no | no | no | no | no | no |  |
| Ground-glass opacities |  | yes | yes | yes | yes | yes | yes | yes | yes | yes |  |
| Consolidation |  | yes | yes | yes | yes | yes | yes | yes | yes | no |  |
| Pleural effusion |  | no | yes | no | no | yes | no | no | yes | yes |  |
| Septal thickening |  | yes | yes | yes | yes | yes | yes | no | no | no |  |
| Percentage of compromised lung |  | >50 | 50 | 50 | >50 | >75 | >50 | >50 | 70 |  |  |
| Deep Vein thrombosis |  | no | no | yes | no | no | no | no | no | no |  |
| Clinical status at 15 days (OMS SCALE) |  | 6 | 4 | 4 | 4 | 6 | 7 | 7 | 5 | 2 |  |
| LV: left ventricle; RV: right ventricle; CTA: Computed tomography angiography; OCT: optical coherence tomography; NA: not available | | | | | |  |  |  |  |  |  |

**Online Table 2. Baseline and Clinical Characteristics of Patients with CT scan positive for pulmonary embolism - Cohort B**

|  | Reference  Value of Range | Patient 1 | Patient 2 | Patient 3 | Patient 4 | Mean ± SD |
| --- | --- | --- | --- | --- | --- | --- |
| Characteristics |  |  |  |  |  |  |
| Age -- yr |  | 58 | 57 | 71 | 72 | 64.5 ± 8.1 |
| Gender |  | male | male | female | female |  |
| Race |  | white | brown | brown | black |  |
| Medical history |  |  |  |  |  |  |
| Conditions |  | Type 2 Diabetes mellitus | no | hypertension | hypertension |  |
|  |  |  |  | paroxysmal atrial fibrillation | hypercholesterolemia |  |
|  |  |  |  |  | depression, fibromyalgia |  |
| Time of symptoms onset at admission -- days |  | 6 | 13 | 12 | 7 | 9.5 ± 3.5 |
| Symptoms |  |  |  |  |  |  |
| Dyspnea / shortness of breath |  | yes | yes | yes | yes |  |
| Dry cough |  | yes | yes | yes | yes |  |
| Fever |  | yes | yes | no | no |  |
| Headache |  | no | no | no | no |  |
| Myalgia / arthralgia |  | no | yes | no | no |  |
| Fatigue |  | yes | no | no | yes |  |
| Type of Mechanical ventilation |  | Non invasive | invasive | invasive | invasive |  |
| Mechanical ventilation -- days |  | 10 | 2 | 4 | 2 | 4.5 ± 3.8 |
| PaO2/FiO2 ratio (mmHg) |  | 86 | 176 | 154 | 121 | 134.2 ± 39.3 |
| Anticoagulation |  | prophylatic | prophylatic | prophylatic | prophylatic |  |
| Laboratory values |  |  |  |  |  |  |
| D-dimer (ng/ml) § | < 500 | 7240 | 14883 | 12899 | 23294 | 14579 ± 6651 |
| C-Reactive protein (mg/liter) | < 5 | 82 | 320 | 542 | 99 | 260 ± 216 |
| Ferritin (ng/ml) | 13 - 150 | 1331 | 3310 | 1678 | NA | 2106 ± 1056 |
| Interleukin 6 (pg/ml) | ≤ 7 | 24 | 5 | NA | NA | 14.5 ± 13.4 |
| Platelet count (10^3^/mm3) | 150 - 400 | 312 | 473 | 257 | 213 | 313.7 ± 113.6 |
| Prothrombin time (INR) | 0.95 - 1.2 | 1.18 | 1.25 | 0.95 | 0.98 | 1.09 ± 0.14 |
| Partial Thromboplastin time (seg) | 25.1 - 36.5 | 15.3 | 24.9 | 47.7 | 28 | 28.9 ± 13.6 |
| White-cell count (10^3^/mm3) | 4 - 11 | 9.3 | 12.5 | 16.6 | 11.1 | 12.3 ± 3.1 |
| Lymphocytes (10^3^/mm3) | 1.5-3.5 | 1.0 | 0.5 | 0.4 | 0.8 | 0.6 ± 0.3 |
| Hemoglobin (g/dl) | >13 | 13.6 | 10.3 | 11.6 | 12.2 | 11.9 ± 1.3 |
| Lactic dehydrogenase (U/liter) | 135 - 225 | 557 | 444 | 742 | 640 | 595.7 ± 126.3 |
| Protein Kinase (U/liter) | <190 | 54 | 1187 | 259 | 1541 | 760.2 ± 716.8 |
| Troponin T (ng/ml) | ≤ 0.014 | 0.010 | 0.084 | 0.093 | 0.029 | 0.1 ± 0.1 |
| NT-proBNP (pg/ml) | <125 | 118 | 473 | 2350 | 630 | 892.7 ± 994.8 |
| Creatinine (mg/dl) | 0.5 - 1.2 | 0.87 | 1.3 | 1.51 | 0.69 | 1.09 ± 0.37 |
| Total bilirrubin (mg/dl) | 0.2 - 1.0 | 0.44 | 2.2 | 0.60 | 0.31 | 0.88 ± 0.88 |
| Echocardiographic findings |  |  |  |  |  |  |
| LV ejection fraction (%) |  | 60 | 60 | 65 | 64 | 62.2 ± 2.6 |
| LV diastolic disfunction |  | impaired relaxation | impaired relaxation | no | impaired relaxation |  |
| RV Dysfunction |  | no | yes | no | no |  |
| Pulmonary hypertension |  | no | NA | no | no |  |
| CTA of chest findings |  |  |  |  |  |  |
| Pulmonary thromboembolism |  | yes | yes | yes | yes |  |
| Ground-glass opacities |  | yes | yes | yes | yes |  |
| Consolidation |  | yes | yes | yes | yes |  |
| Pleural effusion |  | no | no | no | no |  |
| Septal thickening |  | no | yes | yes | yes |  |
| Percentage of compromised lung |  | 35 | 50 | 50 | 50 | 46.2 ± 7.5 |
| Deep Vein thrombosis |  | no | no | no | no |  |
| Clinical status at 15 days (OMS SCALE) |  | 4 | 4 | 4 | 4 |  |
| LV: left ventricle; PH: pulmonary hypertension; RV: right ventricle; CTA: Computed tomography angiography; OCT: optical coherence tomography; NA: not available | | | | | |  |
